# On the Similarity Between the Reinforcing and the Discriminative Properties of Intracranial Self-Stimulation

**DOI:** 10.3389/fnbeh.2022.799015

**Published:** 2022-02-21

**Authors:** David N. Velazquez-Martinez, Benita Lizeth Pacheco-Gomez, Ana Laura Toscano-Zapien, Maria Almudena Lopez-Guzman, Daniel Velazquez-Lopez

**Affiliations:** Departamento de Psicofisiolgía, Facultad de Psicología, Universidad Nacional Autonoma de Mexico, Mexico City, Mexico

**Keywords:** reinforcement, generalization gradient, intracranial self-stimulation, time allocation, pimozide

## Abstract

Rats work very hard for intracranial self-stimulation (ICSS) and tradeoff effort or time allocation for intensity and frequency parameters producing a sigmoidal function of the subjective reward magnitude of ICSS. Previous studies using electrical intracranial stimuli (ICS) as a discriminative cue focused on estimating detection thresholds or on the discrimination between intensities. To our knowledge, there is no direct comparison of the reinforcer tradeoff functions with the discriminative functions. Rats were trained to press and hold the lever for ICSS using the maximum reinforcing intensity below motor alterations or avoidance behavior. First, rats were trained to hold the lever for 1 s; after stability, they undergo trials where intensity or frequency was decreased on 0.1 log step. Thereafter, they undergo further training with a hold of 2 and later of 4 s to determine tradeoff with intensity or frequency. The same rats were trained on a discrimination task where the previously used ICSS signaled a lever where a 1 s hold response was followed by a reinforcing ICSS; on randomly alternating trials, a −0.6 log ICS signaled an alternate lever where a similar hold response led to a reinforcer. After mastering discrimination, generalization tests were carried out with varying intensity or frequency. Rats completed training with 2 and later 4 s hold response. After the completion of each task, the rats had different doses of a pimozide challenge while their intensity and hold-down requirement were varied. With regards to the rats’ tradeoff response time allocation as a function of intensity or frequency, sigmoid functions were displaced to the right when long responses were required. Rats that learned the discrimination task attained a discrimination index of 90–98%. Discrimination accuracy decreased slightly with the increase of hold requirement, but generalization gradients were not displaced to the right as a function of the response requirement. Pimozide induced a dose-dependent displacement of the time-allocation gradients, but it did not affect the generalization gradients. It is concluded that rats integrate response requirements as part of the reinforcement tradeoff function, but the response cost is not integrated into the discriminative function of ICSS.

## Introduction

The intracranial stimulation (ICS) of several regions in the brain has been described to be reinforcing (Olds and Milner, 1954; Olds, 1956; Shizgal, 2001). The stimulation of the medial forebrain bundle at the level of the hypothalamus (MFB-LH) produces the lowest thresholds for intracranial self-stimulation (ICSS) (Gallistel et al., 1981; Shizgal et al., 1989; Wise, 2005; Negus and Miller, 2014). Stimulation trains have several parameters that determine the total current applied and it has been described that, to a large extent, pulse frequency and amplitude may tradeoff to determine the strength of the ICS (Edmonds et al., 1974; Gallistel, 1978; Gallistel and Leon, 1991; Simmons and Gallistel, 1994; Negus and Miller, 2014). The countermodel has posited that subjects integrate amplitude and frequency as a subjective experience of the magnitude of the stimulation described as reinforcer “strength” (Gallistel et al., 1981, 1991; Gallistel and Leon, 1991; Mark and Gallistel, 1993; Simmons and Gallistel, 1994). The curve shift method was introduced to evaluate the relation between reward strength and performance variables (Edmonds and Gallistel, 1974; Miliaressis et al., 1986; Fakhoury and Rompré, 2021) but as discussed by Arvanitogiannis and Shizgal (2008), similar lateral shifts may be produced by alterations in reward probability and cost; therefore, they posited the “Reinforcement Mountain Model” (RMM) as a more precise way to describe the relationship between the commitment to work (allocation time of a particular behavior) to the reinforcer strength and reinforcement rate (Hernandez et al., 2010; Shizgal, 2012). Repeatedly, it has been demonstrated that the amount of work a subject would spend working is related to the joint function of reward strength and opportunity cost (cumulated time or effort to harvest rewards) (Trujillo-Pisanty et al., 2011; Hernandez et al., 2012; Trujillo-Pisanty et al., 2013, 2020; Solomon et al., 2015, 2017).

Considering the functions a stimulus may exert, Skinner (1938, p. 262) posited that “a reinforcing stimulus is at the same time either an eliciting or a discriminative stimulus, but its action in reinforcing a reflex is a separate effect that must be listed among the various functions of stimuli”. Indeed, it has been described that ICS may be used as a conditioned stimulus for an appetitive or aversive unconditioned stimulus (early studies reviewed by Doty, 1969), but pioneer work found that ICS may also serve as a discriminative stimulus for shock avoidance (Nielson et al., 1962) or sucrose reinforcer (Stutz, 1968). A subjective state is produced by ICS and rats can discriminate between its presence and absence (Colpaert, 1977; Colpaert et al., 1977; Phillips and LePiane, 1978), between two different amplitudes (Kornetsky and Esposito, 1981; Druhan et al., 1987a,b, 1989), different frequencies (Nielson et al., 1962; Clincke et al., 1982), or even different loci (Nielson et al., 1962; Hupka, 1970; Wheeling and Kornetsky, 1983, 1984).

Pioneer work comparing the reinforcing and discriminative stimulus of ICS first assured that rats responded for ICSS and then used the previously determined ICSS parameters as a discriminative stimulus for further ICSS (Kornetsky and Esposito, 1981; Wheeling and Kornetsky, 1983, 1984; Schaefer and Michael, 1985, 1988, 1989) of food pellets (Druhan et al., 1987a,b, 1989). It was observed that variations on the ICS current generated differential detection thresholds between the ICS cue and the reinforcing ICS and those thresholds were differentially affected by cocaine (Kornetsky and Esposito, 1981). Further work suggested differential sensitivity of the reinforcing and discriminative stimuli to other pharmacological manipulations; previous work described that while morphine and amphetamine lowered the detection threshold of the reinforcing ICSS, but haloperidol raised it (Marcus and Kornetsky, 1974; Schaefer and Holtzman, 1979; Schaefer and Michael, 1980), the thresholds for detecting ICS as discriminative cue failed to follow similar alterations after the same pharmacological challenges. However, in the latter cases, the reinforcing or discriminative stimuli were evaluated in different sets of animals (Schaefer and Michael, 1985, 1988, 1992). Later, it was found that the effect of haloperidol and amphetamine (Druhan et al., 1987b) or physostigmine (Druhan et al., 1987a) on the ventral tegmental-ICS (VTA-ICS) cue was modulated by trial frequency, but in this case, the ICS was the discriminative stimulus for pellet availability correlated with the low- or high-intensity lever selection.

It should be noted that those studies that used the intensity of the ICS as a cue for further ICSS focused on detection thresholds (presence versus absence) rather than discrimination between ICS amplitudes (Kornetsky and Esposito, 1981; Wheeling and Kornetsky, 1983, 1984; Schaefer and Michael, 1985, 1988, 1989), while those that trained discrimination between ICS amplitudes determined the reinforcing strength with a continuous reinforcement (CRF) schedule but maintained the discrimination with food pellets (Druhan et al., 1987a,b, 1989).

In studies that explored the control over the emission of behavior by interoceptive stimuli, a variety of responses had been used ranging from maze alleys and operant procedures to approach-retract, freezing responses, or conditioned taste aversion procedures (Benoit and Davidson, 1996; Velázquez-Martínez et al., 1999; Davidson et al., 2005; Miranda et al., 2007) but rendering similar generalization gradients of the trained drug dose for most substances. Some studies found that the generalization gradient was displaced to the right by decreasing reinforcer density between blocks of sessions for some drugs (Koek and Slangen, 1982; De Vry et al., 1984; Craft et al., 1998), but others found no differences between schedules of reinforcement for other drugs (Kueh and Baker, 2007). As mentioned previously, decreasing reinforcer density (by increasing work requirement, for example) induced a rightward shift of the function that relates ICSS reward strength to time allocation, therefore, we aimed to determine whether equating reinforcing and discriminative responses as much as possible would produce similar rightward shifts related to increase in effort requirement in the reinforcing and discriminative gradients. Although some reports used a variation on amplitude or frequency as the relevant dimension for a generalization gradient (as described previously), it remains to be shown in the same subjects that variations of the two dimensions produce equivalent generalizations; therefore, we also compared the reinforcing and generalization gradients to variations in amplitude or frequency of the ICS. Finally, we also tested if a challenge with a dopamine antagonist (pimozide) induced similar effects on the reinforcing and discriminative gradients to variations in effort and strength of the ICS (amplitude or frequency), since it has been described that in the case of electrical-ICSS, dopamine neurons are involved in the valuation of opportunity cost but not in the sensitivity of the reward circuitry (Trujillo-Pisanty et al., 2013, 2020).

## Materials and Methods

### Subjects

A total of nine male Long-Evans [Facultad de Psicología, Universidad Nacional Autonoma de Mexico (UNAM)] and five male Wistar rats, 90 days old and weighing 300–350 g at surgery, were individually housed in controlled conditions of temperature and humidity under a normal 12:12 light-dark cycle with a light on at 8 a.m. All rats had continuous access to tap water and a pelleted rodent diet (Rodent laboratory Chow 5001, PMI Nutrition International L.LC., Brentwood, United States). All procedures, housing, and handling observed the National Institutes of Health guidelines for the care and use of laboratory animals (NIH Publications 8th Ed., 2011) and the study had the approval of the Ethics Committee of the Facultad de Psicologia, UNAM.

### Apparatus

The rats were trained in operant conditioning chambers (Lafayette Instruments, Lafayette, IN, United States). One wall of the chamber had a recess for sucrose solution (not used). Retractable levers inserted into the chamber through apertures situated 8 cm above the floor and 5 cm to the right and left of the dispenser could be depressed by a force of approximately 0.2 N. The chambers were enclosed in a sound-attenuating chest with rotary fans and masking noise. A programmable ICSS MED stimulator (PHM-152, MED Associates Inc., Fairfax, VT, United States) provided the train pulses through an electrical swivel (SRO12-0210B10)^1^ and a circular orifice on the roof of the chamber and enclosure. Experimental events and responses were controlled or registered with a MED Associates interface (MED Associates Inc., Fairfax, VT, United States) and a computer located in the same room.

### Drugs

Pimozide (Sigma-Aldrich Chemicals, St. Louis, MO, United States) was dissolved in a 0.3% tartaric acid solution and given in 1.0 ml/kg ip 3 h before the start of the session. Doses (0.031, 0.1, and 0.3 mg/kg) were given in a semi-random order and evaluated twice on each subject and alternated with saline sessions, provided that during the intervening saline sessions, total reinforcers did not vary more than 10% of the pre-administration phase in the time allocation (TA) experiments or that the discrimination index (DI) was above the training criteria (see below). Actually, neither of these criteria were met, so we never skipped an injection session.

### Procedure

#### Surgery

The rats had anesthetic induction with atropine sulphate (Sigma-Aldrich, St Louis, MO, United States) (0.05 mg/kg ip) followed 5 min later by ketamine/xilazine (PISA, Cd. Mexico, Mexico) (87 and 13 mg/kg ip) and maintained under halothane (Sigma-Aldrich, St Louis, MO, United States)/oxygen vapor mixture (0.5–2% halothane); they were positioned in a stereotaxic frame for bipolar electrode (Plastic One, Roanoke, VA, United States) placement aimed at the MFB at the level of the Lateral Hypothalamus (AP: −2.8, ML: ± 1.7, DV: −8.9). The electrode was fixed to the skull with dental acrylic immediately after the surgery rats received antibiotics and diclofenac (8 mg/kg ip). At least 1 week was allowed for surgery recovery before operant training.

#### Behavioral Training

During the first two days of training, the rats received increasing intensities of stimulation of 0.1 ms pulses delivered at 200 Hz in a train of 0.5 s duration to identify the highest intensity below the one that produced any sign of discomfort (motor or freezing) to be used as a reinforcer. Thereafter, the rats were exposed on alternate days to left or right levers with house-light and light above lever turned on during a 30 min session. During such sessions, every 20 s, a tone of 0.5 s duration was accompanied by ICS but any lever press resulted in the immediate delivery of ICSS simultaneous with the 0.5 s tone; this tone accompanied stimulation and was presented whenever rats obtained a reinforcer. After the ICSS train, responses were ineffective for 0.2 s; during this period and during reinforcer delivery lever lights were turned off. Rats that did not learn to press any lever within 3 days, were shaped manually in additional sessions until they obtained at least 100 reinforcers in a 20 min session.

#### Training for Time Allocation

**FIGURE 1:**
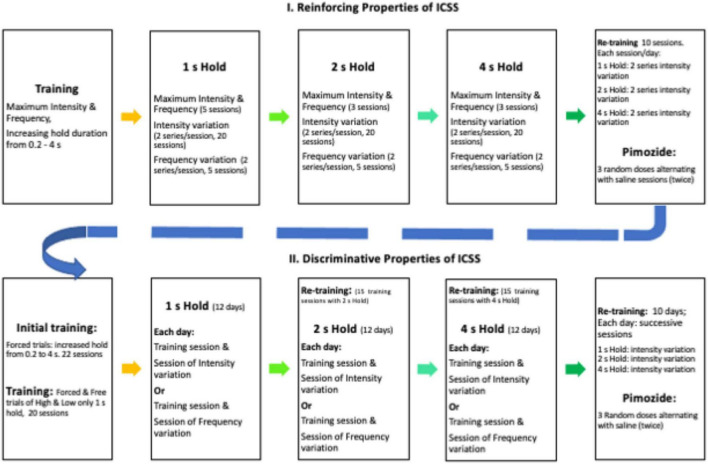
Timeline of experimental conditions (see text for explanation).

Rats were trained in a cumulative hold-down schedule of reinforcement to press and hold the lever depressed for increased durations (0.2, 0.5, and 1 s) for two sessions each duration; only one lever was introduced during any session, and levers alternated daily. Thereafter, the left lever was selected for half of the rats, and the right lever was used for the remaining rats. A series was composed of 15 trials; maximum ICSS intensity and frequency were used during the first three trials; thereafter, six decreasing intensities were used while other parameters held constant; intensities decreased by 0.1 log from the maximum intensity and each decreased intensity was presented randomly (with no replacement) on a trial that alternated with a trial that used maximum intensity. During the initial TA evaluation, each session consisted of two decreasing series; during pimozide and saline evaluation, each session consisted of sox series (2 with 1, 2, and 4 s hold requirement). Each trial was preceded by a priming stimulus equal to the one to be used as reinforcer presented 1 s before the insertion of the lever; trials were separated by 7 s with the lever retracted and the light lever turned off. When the hold responses were required to be 1 s, the trials lasted for 60 s; when the holds were required to be 2 or 4 s, the trials lasted for 120 or 240 s. When the required holds were 1 s or longer, interruptions up to 1 s were classified as TA work as rats typically hold their paw on or above the lever. Upon completion of the trials, the house light was extinguished, and the session ended. The rats were trained with a 1 s hold duration for 20 sessions that took place once a day every day (between 10:00 and 16:00), in the light phase of the daily cycle, 5 days per week. Thereafter, the rats received five sessions where frequencies were varied but all other parameters held constant. On these sessions, the rats received the first three trials with maximum intensity at 200 Hz; thereafter, the frequencies were randomly decreased by 0.1 log steps in trials that alternated with training frequency at maximum intensity. Then, the rats were trained with 2 s (3 sessions of forced trials only, 20 sessions with intensity variations followed by five sessions with frequency variations) and 4 s (3, 20, and 5 sessions as described above) hold requirements. For pimozide evaluation, the rats were retrained during 10 sessions. Each session consisted of two series (of intensity variations) with 1 s, 2 series of 2 s, and 2 series of 4 s hold requirements. Thereafter, the rats received three doses of pimozide injected in random order but alternating with at least one saline injection (see Figure 1 for a timeline of the experimental conditions).

#### Training of Intensity Parameters as Discriminative Stimulus

The same group of rats was trained to discriminate the stimulation used previously as a reinforcer from a −0.6-log stimulus of minor intensity. Briefly, the rats were trained for 10 sessions in the cumulative hold-down (1 s) schedule of reinforcement using the opposite lever used in the TA experiment. Thereafter, sessions started with both levers retracted and the house light extinguished. Then, during high trials, a tone of 1 s that ended with the presentation of the high stimulus (identical to the one used in the TA experiment as a reinforcer) was presented followed by the insertion of the appropriate lever (the opposite lever to that used in the time allocation experiment). The rats had 10 s to complete the hold-down requirement (initially 0.2 s) to receive the reinforcing stimulation (identical to the one used previously in the TA experiment). Levers were retracted immediately after hold-down completion but if there is no response or if the response requirement was not attained, the reinforcer was omitted and the lever was retracted. Even when the hold-down was increased (see below) and the response period was 10 s, the intertrial period comprised the remaining time to 10 s after hold-down completion plus 5 s. In low-trials, the 1 s tone ended with the low stimulus (-0.6 log minor to that used as a reinforcer); then, the opposite lever was inserted and on completion of the hold-down requirement, the reinforcing stimulation was presented (hold requirement, reinforcing stimulation, and accompanying events were identical to those used in the high-trials). Each session was of 150 trials (75 high- and 75 low-trials) and followed the sequence suggested by Colpaert and Koek (1995) for drug-discrimination experiments. During 10 sessions, the rats were exposed to these forced training trials with only the correct lever available for responding; thereafter, hold-down requirement increased to 0.5, 1, 2, and 4 s every three sessions; starting after 1 s, an interruption no longer than 1 s in the hold requirement was considered as working time. During the following 20 sessions, free-choice trials were introduced; on such trials, both levers were inserted after the 1 s tone accompanied by the high- or low-stimulation. The reinforcer followed the response on the appropriate lever, but it was omitted if the initial hold-down was on the incorrect lever. The subject changed their lever-depression after an interruption, or no response was emitted. The proportion of free-choice trials increased until training sessions had 40 high- and 40 low-forced trials and 35 high- and 35 low-free choice trials. Thereafter, generalization sessions were introduced alternating with training sessions provided that free-choice trials had correct responses in at least 80% of high- and low-trials during training sessions. During six generalization sessions, five intermediate intensities (separated 0.1 log apart between high- and low-intensities) were randomly presented between 60 forced trials (30 high and 30 low) and 40 free-choice (20 high and 20 low) trials. On generalization trials, the completion of the hold-down requirement on any lever lead to the reinforcer, but the reinforcer was omitted if there is no response, the hold-down requirement was not attained, or the subject changed their lever-depression after an interruption. The rats underwent another six sessions with generalization trials on which the frequency was randomly decreased by 0.1 log in five steps maintaining the remaining parameters and the anchored low-stimulus parameters constant. Thereafter, the rats were trained for 15 sessions on which the hold-requirement was increased to 2 s, followed by generalization sessions as described above and a further increase in hold-down requirement to 4 s in the training followed by generalization sessions. For pimozide evaluation, the rats were retrained for 10 days where they had three sessions a day with 1, 2, and 4 s hold requirement and free trials with intensity variation (as described previously). Thereafter, the rats received three doses of pimozide injected in a random order but alternating with at least one saline injection, provided that the DIs on the saline sessions were above 80% correct of the free-choice trials (timeline in Figure 1).

### Histology

**FIGURE 2:**
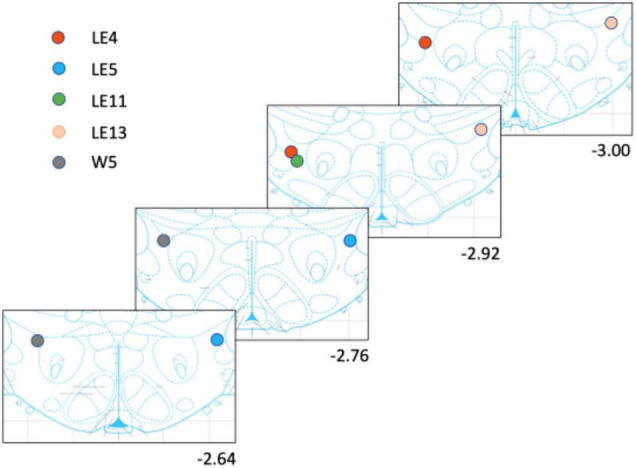
Electrode placement for each rat. The rats had only one implanted electrode either to the right or left side.

The rats were anesthetized (sodium pentobarbital; 200 mg/kg ip) and perfused transcardially using saline followed by 4% paraformaldehyde. The brains were then stored at −80^°^C. Using a vibratome, 40 μm sections were cut to locate the tips of the electrode placement, mounted on glass slides, and stained with blue methylene. Figure 2 shows the tip placements.

### Data Analysis

#### Time Allocation

The first two trials of the session were considered warm-ups and their data were discarded. The data of the last five sessions on each condition were analyzed to obtain cumulated working time in Matlab (MathWorks, Natick, MA, United States). The cumulated working time or TA was expressed as a fraction of the available time during a trial. Since the plots of the averaged TA versus the intensity or frequency variations have a shallower slope than the curves obtained on individual sessions, we fit a function to the TA values for each series variation and averaged its parameters as suggested previously (Hernandez et al., 2006, 2010). Although time allocation varied systematically with intensity or frequency variations on the great majority of the trials, some aberrant cases were observed. We filtered the data by averaging contiguous time allocations of trials that were higher than the next higher intensity or frequency (no more than 2% of all trials). A four-parameter equation was fitted to the data: (TA − TAmin)/(TAmax − TAmin) = 1/[1 + exp{−Slp × (log10 (d) − Loc)}]. Where: **d** = Pulse intensity or frequency, **Loc** = Value of the location parameter (it refers to the placement of the curve within the stimulation range, and together with TA_50_ informs about rightward or leftward shifts), **TA** = Time allocation, **TAmin** = Minimal time allocation, **TAmax** = Maximal time allocation, and **Slp** = Slope parameter determining the steepness of the rise (Trujillo-Pisanty et al., 2020; Pallikaras et al., 2021). PRISM (v9, GraphPad Software, San Diego, CA, United States) was used to fit functions, statistical analysis, and produce data graphs.

#### Intracranial Stimuli Discrimination

Proportion of reinforces on choice trials obtained by responding to the lever correlated with the high-intensity stimulus were used to estimate discrimination indices (DI_Sr); we also used the proportion of the first hold-down response (even if the rat did not complete the requirement of changed lever after an interruption) to estimate the DI_Hld. Therefore, the DIs were estimated as the proportion of reinforcer DI_Sr or holds DI_Hld obtained by responding to the lever correlated with the high-stimulus after each stimulus value to the total number of trials where that particular stimulus value was presented; thus, a DI of 1 meant that all reinforcers (or hold) were obtained by responding toward the high-stimulus value; conversely, a DI = 0 meant that all reinforcers (or holds) were obtained by responding to the low-intensity lever, while a DI = 0.5 meant random choice after the ICS stimulus. The same equation for time allocation was used to fit the discrimination data: (DI − DImin)/(DImax − DImin) = 1/[1 + exp{−Slp × (log10 (x) − Loc)}]. **d**, **Loc,** and **Slp** had the same meaning while **DI** = Discrimination Index, **DImin** = Minimum DI, and **DImax** = Maximal DI. As before, we averaged its parameters to obtain curve fitting for intensity and frequency variation and DIs. To compare the performance of the correct responses on generalization trials, we used two-way repeated measures using the statistical package of PRISM.

#### Comparison Between Time Allocation and Discrimination Index

To determine the relationship between intensity and frequency variation we converted all frequency and intensity variations to a common scale by multiplying amplitude (μA) * pulse duration (0.1 ms) * pulses (Hz) in train (0.5 s) (see Gallistel and Leon, 1991, p. 921). Also, to compare TA_50_ ([TAmax − TAmin]/2) and DI_50_ ([DImax − DImin]/2) and since rats were exposed to different current intensities, we expressed TA_50_ or DI_50_ as a fraction between max and min by anchoring the minimum intensity to 0 and the maximum intensity to 1 with the following transformation: f(x) = ([TAmax − TAmin]/[1 + exp{−Slp(log10[×{max_x_−min_x_} + min_x_] − Loc)}]) + TAmin, where max_x_ = maximal stimulation strength in common units, and min_x_ = minimum stimulation strength in common units; all other terms as described above. We substituted DI-parameters instead of TA-parameters when ICS was used as a discriminative cue. To compare the similarity between the TA and DI functions, we plotted the TA_50_ against DI_50_ assuming that if subjects incorporate response cost as one of the properties to discriminate, the DI_50_ will increase following the increase in response requirement and will find an orderly relation between Tas and Dis; if the subjects do not incorporate the response cost, Tas and Dis will be unrelated.

## Results

### Time Allocation Performance

The 9 Long Evans and 5 Wistar rats completed the TA evaluation (training and evaluation of frequency and amplitude variations). However, five rats developed a strong preference for the lever that delivered reinforcers during the TA experiment and therefore, their DIs were unreliable and four rats lost their electrode before completing all the evaluations of the discrimination experiment; therefore, for all these rats, we present their TA plots in Supplementary Material but we omitted them in the presentation of the following results.

**FIGURE 3:**
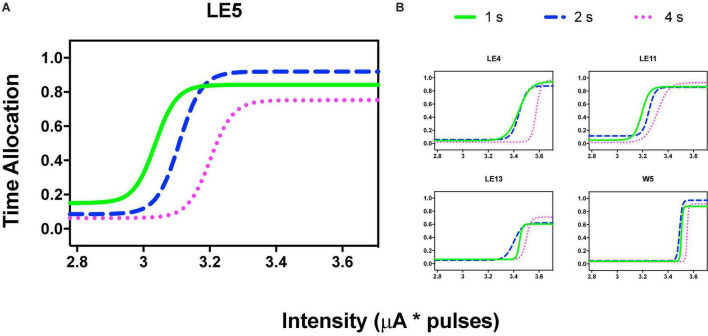
Time allocation as a function of variations of intensity (in common scale, see text) and price (hold requirements). Abscissas in log scale. **(A)** Exemplar rat. **(B)** Remaining rats.

**FIGURE 4:**
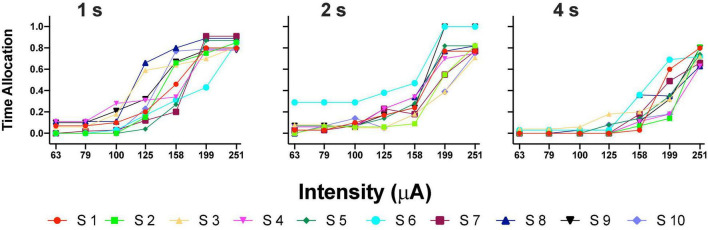
Time allocation as a proportion of trial duration in each trial series. Plots in Figure 3 were obtained from the averaged parameters of individual plots.

**FIGURE 5:**
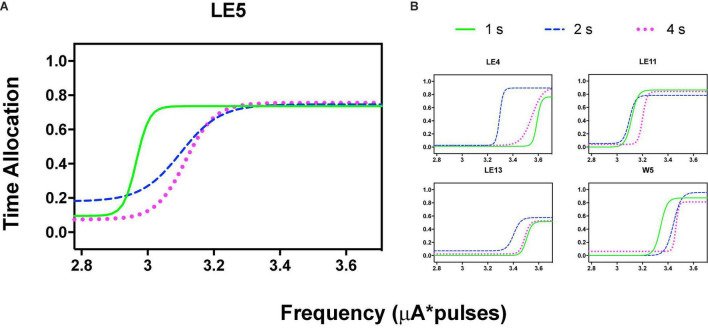
Time allocation as a function of variations of frequency (in common scale, see text) and price (hold requirements). Abscissas in log scale. **(A)** Exemplar rat. **(B)** Remaining rats.

Figure 3A shows the TA after variations of amplitude for an exemplar rat; note that abscissas use the common scale for amplitude and frequency. As presented in the data analysis, each plot of Figure 3 is the result of the averaged parameters of the fitted function to each series variation; Figure 4 shows an example of the individual series of the proportion of the TA to the total trial duration after the intensity variations and hold down requirements; Table 1 presents the mean goodness of fit from all series that correspond to each plot of Figures 3, 5. Supplementary Figure 1 shows the plots using μA units and Supplementary Figures 2A,B show the corresponding plots for the rats that did not learn the ICS discrimination or lost electrode before completion. We did not use the “Reinforcement Mountain” plot style because we only had three values of reinforcement cost, we did not collect trial-to-trial variations of hold-down requirements, and we did not collect radial variations (simultaneous variations in strength and hold requirement). As shown for rat LE5, increasing the hold-down requirement from 1 to 4 s produced a rightward shift of the function that relates to the reinforcer strength and TA. In three rats, the curves for 1 and 2 s TA were quite close, but the curves for 2 and 4 s were orderly displaced from left to right. Saturation by the reward stimulation at high frequency and amplitude may induce the rats to space their responses to avoid aversive effects (see Discussion) and may be related to the closeness of the 1 and 2 s curves. It should be noted that in most of the remaining rats (Supplementary Figures 2A,B), curves were orderly displaced to the right, consistent with the predictions of the RMM.

**TABLE 1:** Mean goodness of fit of all series that correspond to each plot of TA.

Time allocation: goodness of fit (R squared)						
	μA			Hz		
Subject	1 s	2 s	4 s	1 s	2 s	4 s
LE4	0.992 ± 0.004	0.998 ± 0.002	0.994 ± 0.004	0.971 ± 0.015	0.993 ± 0.003	0.997 ± 0.001
LE5	0.993 ± 0 03	0.999 ± 0.028	0.972 ± 0.016	0.991 ± 0.004	0.995 ± 0.001	0.992 ± 0.003
LE11	0.985 ± 0.006	0.989 ± 0.003	0.982 ± 0.008	0.991 ± 0.004	0.994 ± 0.003	0.996 ± 0.002
LE13	0.978 ± 0.009	0.978 ± 0.008	0.952 ± 0.021	0.964 ± 0.028	0.998 ± 0.001	0.988 ± 0.005
W5	0.981 ± 0.009	0.992 ± 0.008	0.993 ± 0.003	0.986 ± 0.011	0.999 ± 0.001	0.989 ± 0.004

Orderly rightward shifts after the variations in stimulation frequency are depicted in Figure 5A for the same rat and Figure 5B shows the remaining rats, all plotted in the common scale. Supplementary Figures 3, 4 show the plots of all rats. As with the variation in amplitude, some rats showed extreme rightward shift after 1 s hold-down requirement; consistent with the saturation explanation (see Discussion) all rats showed an orderly rightward shift from 2 to 4 s TA.

**FIGURE 6:**
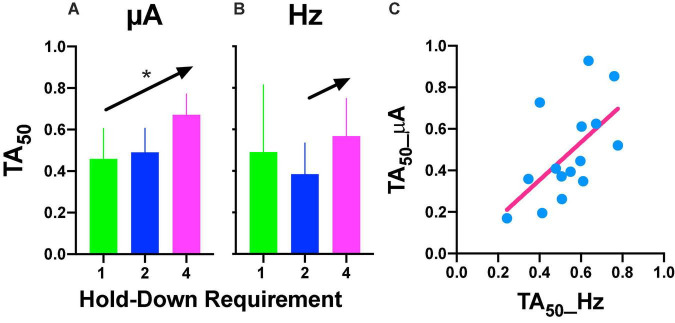
**(A)** Time allocation (TA)_50_ after intensity or frequency **(B)** variations. Mean ± SE (5 subjects) and arrows and * show a significant trend between the means. **(C)** Relationship between TA_50_ observed after variations in frequency or intensity. Each point is the TA_50_ of each rat plotted in common units (see text) at different prices (hold-down requirement).

Repeated measures one-way ANOVA confirmed significant [*F*_(2_, _8)_ = 22.89, *p* = 0.005] differences between the hold-down requirements and a significant [*F*_(1_, _8)_ = 39.29, slope 0.106, *p* = 0.002] trend between the hold-requirements after intensity variations (Figure 6A). However, if we include the data of the 1 s hold (subjects with the extreme rightward shift, see discussion), no significant effects or trend was observed for frequency variations (Figure 6B); however, a one-tailed repeated measures *t*-test of only 2 and 4 s hold requirement revealed significant differences [*T*_(4)_ = 2.499, *p* = 0.033]. To determine the similarity between the frequency or intensity variations we plotted individual TA_50_ intensity against TA_50_ frequency variations after each price used (Figure 6C). Although the dispersion of individual TA_50_s (R squared = 0.36) was large (in relation to those subjects that presented the extreme rightward shift), the slope of the regression line was t 0.909, suggesting the confirmation of the tradeoff between amplitude and frequency.

### Stimulus Control by Intracranial Stimuli

As mentioned, some rats developed an exclusive preference for the lever where TA was evaluated. Five rats were able to learn the discrimination and achieved DIs larger than 90% accuracy after either the high- or low-current stimuli; therefore, almost mirror plots were obtained when we expressed the DIs as a fraction of the responses to the low- or high-intensity stimulus lever selection (not shown). Also worthy of comment, is that rats completed almost all choice trials of low- or high-amplitude; Supplementary Figure 5 depict missed trials (those that had no response to either lever out of 20 low- or 20 high amplitude trials); as shown, on average no more than two trials were missed per session and repeated measures two-way ANOVA confirmed no significant differences between high- or low-intensity levers [*F*_(1_, _4)_ = 0.336, *p* = 0.592], prices [*F*_(2_, _8)_ = 1.046, *p* = 0.394], or interaction [*F*_(2_, _8)_ = 2.651, *p* = 0.130].

**FIGURE 7:**
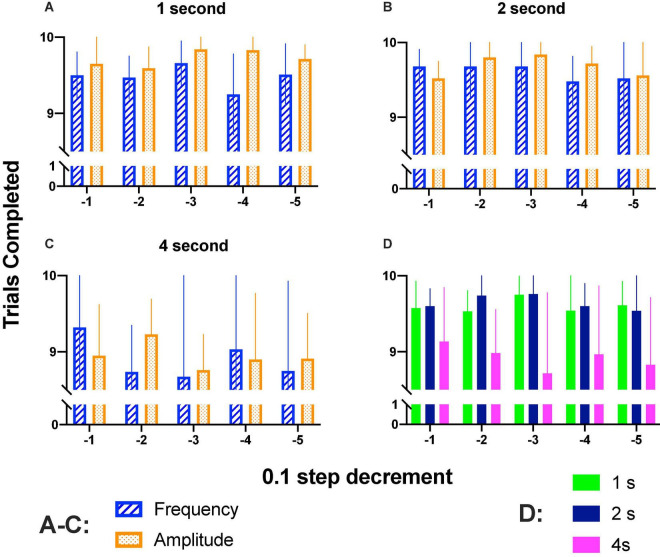
**(A–C)** Mean completed trials (10 of each parameter variation) when increased (1–4 s) hold-down durations were required. **(D)** Completed trials (combined intensity and frequency variations) for 1–4 s hold down requirement. Mean and SE of five subjects per bar.

We wondered whether rats completed trials along the step size decrements; Figures 7A–C show that rats completed most trials during generalization tests. Repeated measures two-way ANOVA confirmed that there were no significant differences between step sizes [1 s: *F*_(4_, _16)_ = 0.863, *p* = 0.506; 2 s: *F*_(4_, _16)_ = 0.890, *p* = 0.492; 4 s: *F*_(4_, _16)_ = 0.355, *p* = 0.836]; parameter variation [1 s: *F*_(1_, _4)_ = 1.777, *p* = 0.365; 2 s: *F*_(1_, _4)_ = 2.222, *p* = 0.210; 4 s: *F*_(1_, _4)_ = 0.0486, *p* = 0.836] or interaction [1 s: *F*_(4_, _16)_ = 1.119, *p* = 0.382; 2 s: *F*_(4_, _16)_ = 0.571, *p* = 0.687; 4 s: *F*_(4_, _16)_ = 0.870, *p* = 0.502].

Completed trials as a function of hold-down requirement are presented in Figure 7D; as shown, on session where the hold-down requirement was of 4 s, there was a trend to complete fewer trials; repeated measures ANOVA confirmed significant differences between prices [*F*_(2_, _8)_ = 14.5, *p* < 0.002] but no differences between step decrement [*F*_(4_, _16)_ = 0.206, *p* = 0.931] or interaction [*F*_(8_, _32)_ = 0.584, *p* = 0.782]. As mentioned in the data analysis, we estimated DIs based on reinforcers (S^R^) (Supplementary Figures 6, 7) or hold-downs emitted (see below). For amplitude variations, DI_50_-S^R^ or DI_50_-Hold for most rats were proportional (Supplementary Figure 8) but the main difference between the two procedures to estimate DIs was that with the holds, we observed higher DIs at the high-amplitude or high-frequency of the generalization gradient as compared to DI-S^R^. The decrement of the completed trials observed may explain the lower DIs observed with DI-S^R^. As even a single hold-down to the correct lever reveals appropriate discrimination, we preferred to use DIs-hold than DI-S^R^.

**FIGURE 8:**
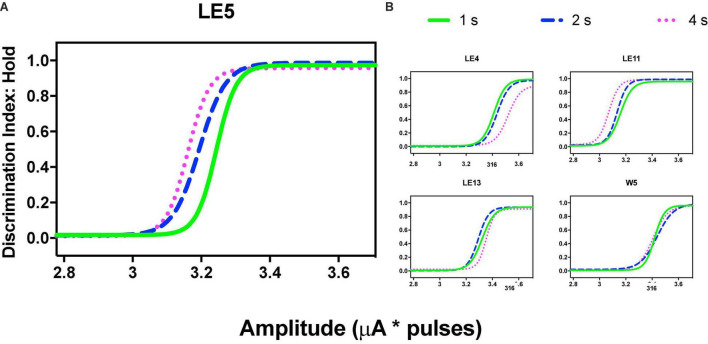
Discrimination Index as a function of variations of intensity (in common scale, see text) and price (hold requirements). Abscissas in log scale. **(A)** Exemplar rat. **(B)** Remaining rats.

Using the common scale, all rats showed orderly gradients after variations of intensity; these plots were obtained by averaging the parameters after fitting the function (see section “Data Analysis”) to each series, and Table 2 presents the mean goodness of fit to each series. Figure 8A shows the same rat depicted in Figures 3A, 5A of TA while TA plots showed rightward shifts as the price increased. DIs showed an inverse relation: as the price increased, we observed a trend toward a leftward shift instead of the rightward shift of TA. Rat LE11 showed a similar trend, but the DIs of the other rats showed minimal effects of the price increments (Figure 8B).

**TABLE 2:** Mean goodness of fit of all series that correspond to each plot of generalization.

Discrimination: goodness of fit (R squared)						
	μA			Hz		
Subject	1 s	2 s	4 s	1 s	2 s	4 s
LE4	0.994 ± 0.001	0.992 ± 0.003	0.980 ± 0.008	0.962 ± 0.009	0.984 ± 0.004	0.962 ± 0.014
LE5	0.986 ± 0.009	0.979 ± 0.008	0.991 ± 0.002	0.995 ± 0.002	0.992 ± 0.003	0.993 ± 0.002
LE11	0.988 ± 0.002	0.992 ± 0.002	0.995 ± 0.002	0.982 ± 0.008	0.984 ± 0.006	0.978 ± 0.009
LE13	0.977 ± 0.007	0.989 ± 0.003	0.973 ± 0.008	0.991 ± 0.003	0.982 ± 0.005	0.990 ± 0.009
W5	0.991 ± 0.002	0.973 ± 0.008	0.986 ± 0.003	0.976 ± 0.006	0.988 ± 0.001	0.991 ± 0.005

**FIGURE 9:**
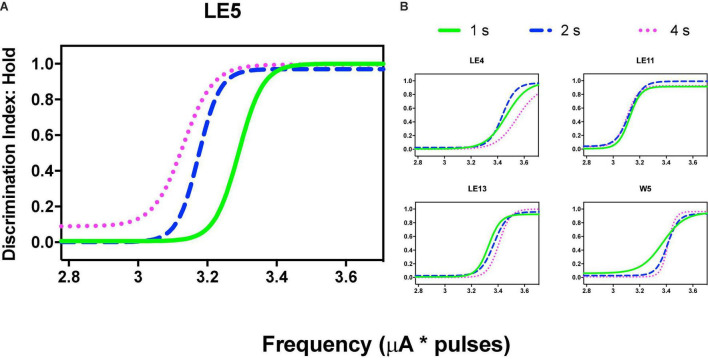
Discrimination Index (DI) as a function of variations of frequency (in common scale, see text) and price (hold requirements). Abscissas in log scale. **(A)** Exemplar rat. **(B)** Remaining rats.

Variations in frequency produced orderly generalization gradients; only rat LE5 produced notorious leftward shifts as the price increased (Figure 9A) but price increases did not alter the generalization gradients of the other rats (Figure 9B).

### Comparison Between Reinforcement and Stimulus Control Gradients

**FIGURE 10:**
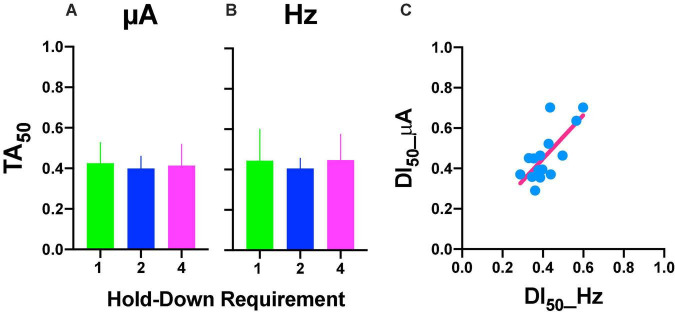
DI_50_ after intensity **(A)** or frequency **(B)** variations. Mean ± SE (5 subjects). **(C)** Relationship between DI_50_ observed after variations in frequency or intensity. Each point is the DI_50_ of each rat plotted in common units (see text) at different prices (hold-down requirement).

Repeated measures one-way ANOVA yield no-significant [*F*_(2_, _8)_ = 1.777, *p* = 0.227] differences between hold-down requirements and a no-significant [*F*_(1_, _8)_ = 0.056, slope −0.007, *p* = 0.819] trend between hold-requirements after intensity variations (Figure 10A). Also, we observed no-significant [*F*_(2_, _8)_ = 0.219, *p* = 0.808] differences between hold-down requirements and a no-significant [*F*_(1_, _8)_ = 0.002, slope 0.002, *p* = 0.964] trend between hold-requirements after frequency variations (Figure 10B). To evaluate the similarity between frequency or intensity variations, we plotted individual DI_50_ intensity against DI_50_ frequency variations (Figure 10C). Worth of notice is despite having no trends in the hold-down requirements (that means the price does not influence discrimination), there was a high correlation for each rat between its DI_50_ after the intensity and frequency variations, as shown by the slope (1.083) of the regression line, confirming the tradeoff between amplitude and frequency with lower dispersion of individual DI_50_s (R squared = 0.53) than TA_50_ (Figure 6C).

**FIGURE 11:**
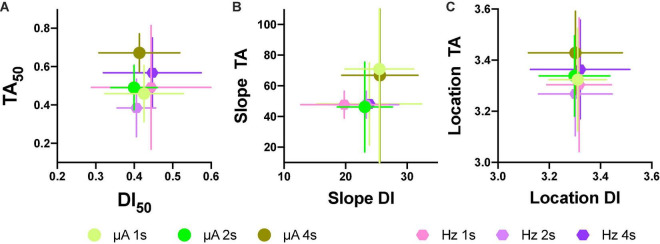
Individual relations between TA50 to DI50 after intensity or frequency variations **(A)**. Relation to mean slope **(B)** and location parameters **(C)** after intensity or frequency variations (each point *N* = 5 ± S.E.). Horizontal error bars are for DIs, while vertical error bars are for TAs.

Based on the generalization gradients, we observed that the stimulus properties of the ICS differed from the reinforcement gradients of the ICSS. To confirm these observations, we plotted mean TA_50_ against mean DI_50_ for both intensity and frequency (Figure 11A; individual plots are in Supplementary Figures 9A,B) and mean slopes (Figure 11B) and location (Figure 11C) parameters. Since rats differed on the maximum stimulus intensity, TA or DI were expressed as a fraction of the maximum stimulation used while the common scale was used to estimate the slope and location parameters. As shown, no consistent relationship between TA and DI emerged; while large changes in TA_50_ were observed as the price increased (increase in color darkness seen in the vertical direction), almost no variations in DI_50_ occurred related to price variations (note scale and no displacement in the horizontal axis in plots of Figure 11). Slopes were larger for TA than DI (see differences in axes) and had no consistent variation in relation to price (see Supplementary Figure 9 for individual plots). Location parameters varied for TA in relation to the hold duration (displacements in the vertical direction) but had no variation for Dis (Horizontal direction).

### Effects of Pimozide Upon the Reinforcement and Generalization Gradients

Each rat received all doses of pimozide two times after completion of the TA and after completion of the generalization experiments. Reinforcement or generalization gradients were obtained after variations in amplitude and prices. Location and slope parameters were obtained using common units, but TA_50_ or DI_50_ are expressed relative to the maximum stimulation used as a reinforcer or discriminative cue; in the case of three rats, even with the largest intensity and frequency used, the 0.3 mg/kg dose decreased responding, so their TA_50_s were estimated by extrapolation. It is worth noticing that in the generalization procedure, the rats responded and we were able to estimate their DI_50_s without extrapolation. Although all animals used completed the TA pharmacological challenge, data analysis included only the five rats that also completed the pharmacological challenge with the generalization procedure and therefore, pimozide data should be considered as preliminary data and is presented in Supplementary Figure 10. When the TA data were analyzed with a two-way repeated-measures no significant differences emerged for the dose factor [*F*_(3_, _12)_ = 2.334, *p* = 0.127], price factor [*F*_(2_, _8)_ = 2.967, *p* = 0.1087], or interaction [*F*_(6_, _24)_ = 1.187, *p* = 0.346]. For the slope of the TA function, no significant differences emerged for the dose [*F*_(3_, _12)_ = 1.725, *p* = 0.215], price [*F*_(2_, _8)_ = 0.818, *p* = 0.475], or interaction [*F*_(6_, _24)_ = 0.483, *p* = 0.814]. Also, in the case of location there were no significant differences for the dose [*F*_(3_, _12)_ = 2.790, *p* = 0.086], price [*F*_(2_, _8)_ = 1.487, *p* = 0.282] or interaction [*F*_(6_, _24)_ = 1.125, *p* = 0.377]. Worth of notice is that one-way repeated measures ANOVA for the TA_50_ saline condition showed significant differences [*F*_(2_, _8)_ = 16.36, *p* = 0.001] between hold-down requirements and a significant trend [*F*_(1_, _8)_ = 31.09, *p* = 0.001] that reproduced the effect of price increase on TA plotted in Figure 6A.

In the case of the DI_50_ of the generalization gradient, we found no significant differences for dose [*F*_(3_, _12)_ = 1.651, *p* = 0.230], price [*F*_(2_, _8)_ = 1.394, *p* = 0.302], or interaction [*F*_(6_, _24)_ = 0.8147, *p* = 0.569]. For the slope, we found a significant effect of dose [*F*_(3_, _12)_ = 6.334, *p* = 0.008], but not price [*F*_(2_, _8)_ = 0.909, *p* = 0.440] or interaction [*F*_(6_, _24)_ = 0.960, *p* = 0.472]. In the case of the location parameter, no significant differences for dose [*F*_(3_, _12)_ = 1.554, *p* = 0.251], price [*F*_(2_, _8)_ = 0.990, *p* = 0.412], or interaction [*F*_(6_, _24)_ = 0.980, *p* = 0.459] were observed.

Although the variability between subjects and the limited number of rats prevented us to find statistical differences for the preliminary data, consistent effects were observed. Pimozide induced a dose-dependent leftward shift (seen as an upward movement of TA_50_) of the reinforcement gradient (Supplementary Figure 10A); the shift of the gradient was also dependent on the response requirement since at each dose, the leftward shift at 4 s requirement was larger than at 1 or 2 s requirement. However, pimozide induced no displacements of the generalization gradients (Supplementary Figure 10D). Consistent with the rightward displacements of TA_50_, the location parameter increased for TA (Supplementary Figure 10C), but not for the generalization gradients (Supplementary Figure 10F). At the 1–2 s hold-down requirements, pimozide increased TA slope, but it decreased DI slopes (note scales of Supplementary Figures 10B,E).

## Discussion

We aimed to determine whether reinforcement and generalization gradients are similar and whether rats incorporate response effort as part of the cue signal in the discrimination of stimulation strength. First, we were able to reproduce previous research that showed rightward shifts of reinforcement gradients as effort requirement increased. Then, using the same reinforcing stimulus as a cue for lever selection, we were able to observe orderly generalization gradients as a function of stimulation strength. However, differences emerged between reinforcement and generalization gradients. The slopes of the reward gradients were steeper than those of the stimulus generalization functions. More importantly, while rats take cost-benefit as a determinant of their reinforcement gradient, it seems that they exclude cost-benefit from their generalization gradients. Although the small number of subjects complicated the statistical analysis, the experiment may also be conceptualized within the framework of single-subject research (Sidman, 1960), each subject replicating the effect of the experimental manipulations; within this framework, quite consistent effects of manipulations were observed.

Early studies described that rats can detect variations of stimulation parameters and were able to adjust their response rate in accordance with the reinforcer strength (Wauquier and Niemegeers, 1972; Koob, 1977). One of the earliest suggestions was that the reinforcer strength determines TA to a platform and comes from the observations of Valenstein and Meyers (1964), but the RMM is the more elaborated model to describe the relationship between the commitment to work or TA to reinforcer strength and reinforcement rate (Arvanitogiannis and Shizgal, 2008; Shizgal, 2012). Although our experimental design with only three different prices precluded us to fit the RMM to our data, still, we were able to show that the reinforcement gradient had a leftward shift as the work requirement increased from 1 to 4 s. In some rats, we were unable to see a shift from 1 to 2 s requirement (see below), but in all rats, we observed a clear leftward shift with a 4 s requirement. These observations are consistent with the RMM.

As mentioned, it has been posited that subjects integrate the amplitude and frequency of the ICS as a subjective experience described as reinforcer “strength” (Gallistel et al., 1981, 1991; Gallistel and Leon, 1991; Mark and Gallistel, 1993; Simmons and Gallistel, 1994); in accordance, we observed orderly decrements of TAs or DIs as we decreased intensity or frequency. Using a titration procedure where variation in one option (e.g., amplitude) offsets the variation in the alternative option (e.g., frequency), Gallistel and Leon (1991) found that the slopes of the regression line for pulse and current were quite similar between a fixed strength option and the titration procedure in the alternative option allowing a moment-to-moment comparison between the two parameters and a reduction of variability remembrance when comparing the available options. In our case, in both the time-allocation and discrimination procedures, the comparison between intensity and frequency variations were carried out in successive sessions, introducing remembrance variability as shown by the dispersion of TA_50_s or DI_50_s around the regression line; despite such inconvenience, slope lines relating frequency to intensity were very close to 1 (TAs: 0.909, DIs: 1.083) as predicted for perfect substitutability (Gallistel and Leon, 1991).

As mentioned before, several studies were able to train ICS as a discriminative stimulus for further ICSS (Kornetsky and Esposito, 1981; Wheeling and Kornetsky, 1983, 1984; Schaefer and Michael, 1985, 1988, 1989) of food pellets (Druhan et al., 1987a,b, 1989). Early attempts to compare the reinforcement and the generalization gradient identified high- and low- rewarding intensities to be used as discriminative cues, but the cues signaled food pellets correlated with lever-selection with no reinforcement available during the generalization trial (Druhan et al., 1987a,b, 1989), thus, although the ICS was reinforcing and used as a discriminative cue, such functions occurred at different times. In the case of Kornetsky and Esposito (1981), the ICS was used as a cue for a further ICSS (as in the present study) but they focused only on the detection threshold for the reinforcing and cue function. In the present study, it seems that the ICS exerts a strong discriminative control for a further ICSS producing accurate lever selection correlated with the variations of strength in the discriminative cue. Since the TA and the discrimination procedures had the same response requirements and similar stimulation parameters were used, an accurate comparison between the reinforcement and generalization gradient was guaranteed.

It should be noted that we used the highest stimulation strength supported by the subject delivered at 200 Hz, just below the one that would produce motor effects. We choose such parameters to maximize the probability that a 0.6 log decrement of amplitude would still be sensed by the rat. Although we do not have direct evidence (we did not explore the threshold curve first) that a 0.6 log decrement in amplitude from maximal strength may produce a subjective experience in our rats, some evidence from previous work suggests that our stimulation parameters were within the stimulus range that rats can detect. For reinforcing stimulation, it was suggested (Fakhoury and Rompré, 2021) to start training with a 400 ms train of 0.1 ms pulse at 200 μA and 75 Hz and increase the reinforcer strength in 0.1–0.2 log units. When we varied stimulation strength, we decreased amplitude or frequency, leaving the other at its maximum, and therefore, our reinforcer parameters were within the range that rats could detect. Therefore, when confronted with low reinforcer strength, the rat may choose not to respond because such magnitude of the reinforcer was not worth the effort. In the case of stimulation as a discriminative cue, while the stimulation parameters reported are not directly comparable to ours, one study (Phillips and LePiane, 1986) found that rats started stimulation detection at 12 μA with a 200 ms sine wave train at 0–28 μA and 66 Hz; others found detection from 8 to 12 μA with four pulses separated from the preceding one by 200 ms (Druhan et al., 1987b,^1989^) or with several trains separated by 20 s during a 2 min period (Druhan et al., 1987a,b) of a 60 Hz sine wave. Another study (Kornetsky and Esposito, 1981), with more similar parameters than ours (500 ms train of 0.2 ms pulse duration at 60 Hz), found a threshold of 65–125 μA for reinforcement and 7–22 μA for stimulus detection. In line with these findings, our rats missed a similar number of trials with the low than with the highest amplitude and neither was observed that rats differentially missed trials along with the intermediate durations. Also, a mirror gradient would be obtained if we plot the generalization gradient using the proportion of holds to the lower amplitude stimulus cue. Although at this moment, we cannot exclude that discrimination was based between presence versus absence of stimulation and we have no direct proof of the subjective experience, behavioral data of strong stimulus control suggest cue detection.

An inconvenience of the use of the strongest current supported by the subjects is that we approached saturation (Gallistel et al., 1991; Gallistel and Leon, 1991; Simmons and Gallistel, 1994; Leon and Gallistel, 1998; Sonnenschein et al., 2003; Solomon et al., 2015) of the reward current and, as Gallistel and Leon (1991) point out, “the subjective representation of the rate of reward is proportionate to the objective rate and combines multiplicatively with the subjective magnitude of reward to determine the subjective net rate of reward … [but] the approximation obviously fails when the effect of a stimulation parameter on reward magnitude saturates”. In the case of some rats, performance with 1 s of hold-down was shifted to the right and got a lower maximum asymptote than with hold-down of 2 and 4 s; A 1 s hold-down may have produced too short an interval between pulse trains with high frequency and high current that the effective charge could be too high to produce saturation and broken down the memory of the magnitude of past reward and desire to get more reward inducing variability in the plots that relate TA_50_s or DI_50_s after amplitude or frequency variations. Also, more dispersion in the TA_50_s than in the DI_50_s around the regression lines is in accordance with the suggestion that performance measures could be more affected by the saturation of reward effects than underlying subjective effects (Gallistel et al., 1991). An alternative worth considering is the finding that subjective prices converge to objective prices beyond 3.18 s; below such lower bound, prices may preclude an accurate evaluation of alternate activities to TA (Solomon et al., 2017) and therefore, TA would not follow accurately very low priced reinforcers. To disentangle these options, larger variations of response requirements would be explored.

The main finding is that the reinforcement gradient incorporates the effort required to obtain reinforcers while the generalization gradient was insensitive to it. We manipulated effort as a hold-down requirement, while some previous experiments of drug discrimination used an indirect assessment of effort since they evaluated whether schedule requirement altered the generalization gradient. Preliminary evidence suggested that rats may incorporate the response cost of the discriminative stimulus in morphine—saline (Craft et al., 1998) or saline—fentyl discrimination (De Vry et al., 1984) since they observed a leftward shift in generalization gradient after an increase in reinforcer density for the drug correlated lever; however, rather than discuss that such displacement was related to effort, they suggested that response feedback was used as a cue for the discrimination (see also Koek and Slangen, 1982). Later, it was suggested that the reinforcement schedule had little influence on stimulus generalization between MDMA and cocaine (Kueh and Baker, 2007). In the case of ICS, Schaefer and Michael (1992) found no changes in the detection thresholds for ICSS in an ICS discrimination (versus its absence) despite the drugs inducing large increases in locomotor activity and responding for ICSS. Our study agrees with such early suggestion but, while differential reinforcer densities may have occurred between reinforcement schedules or drugs may have induced alterations in response topography that would induce differential cues for discrimination. In our case, there were no such confounding variables since responses were similar between TA and the response requirement of the discriminated response. Therefore, our results strongly suggest that while cost was a prime determinant for the reinforcement gradient, it does not influence it. An alternative suggestion would be that as effort increases, rats pay more attention to the discriminative cue (as in rats LE5 and LE11); when they are not sure about the discriminative cue, their best strategy would be not to engage in a high-cost response. This would explain the leftward shift and the increased number of trials missed as hold requirement increases, but would require a proper experiment to prove it.

Early reports described that pimozide decreased responses were maintained by ICSS; of these reports, several suggested that response reduction was related to the output of motor behavior (Wauquier and Niemegeers, 1972) or reduced reward effectiveness (Liebman and Butcher, 1974; Zarevics et al., 1977; White et al., 1978; Wauquier, 1979; Ettenberg and White, 1981). Consistent with reduced reward effectiveness, pimozide reduced extinction responses previously maintained with ICSS (Ettenborg et al., 1979), increased reinforcer thresholds using a titration procedure (Zarevics and Setler, 1979), and produced a rightward shift of a reinforcement gradient after variations in amplitude (Phillips and LePiane, 1986). However, others suggested that pimozide reduced responding for ICS by decreasing response initiation or maintenance rather than an effect on the rewarding process (Fibiger et al., 1976); to a similar conclusion, others arrived at and observed that pimozide did not maintain response rate for ICSS after initially responding to the lever (Fouriezos and Wise, 1976; Fouriezos et al., 1978). As mentioned previously, response rate measures or even the curve shift method introduced to evaluate the relation between reward strength and performance variables (Edmonds and Gallistel, 1974; Miliaressis et al., 1986; Fakhoury and Rompré, 2021) had limitations because similar lateral shifts may be produced by alterations in reward strength and cost (Arvanitogiannis and Shizgal, 2008). Using the RMM to fit the effects of pimozide pretreatment on the proportion of time allocation working for ICSS as a function of pulse frequency and hold-down requirement (price) described that pimozide did not alter the sensitivity of brain reward circuity but changed the reward-system gain, subjective effort costs, and/or the value of activities that compete with ICSS (Trujillo-Pisanty et al., 2013). The present data, although preliminary, are consistent with such suggestion. Pimozide produced a dose-dependent rightward shift of reinforcement gradients, and the displacement increases with the response requirement (price).

In the pioneering work that compared the reinforcing and discriminative functions of ICSS, it was observed that pimozide pretreatment selectively decreased the response rate for the reinforcing effect of ICSS but had no such effect on the detection threshold for the ICSS cue (Bird and Kornetsky, 1990) or on the discriminated responses in mice (Bowers et al., 1985). Present results confirm and extend such reports. In those reports, the discriminative function was explored using a different schedule of reinforcement than that used to evaluate the reinforcing function; therefore, response feedback may underline differences between reinforcer and discriminative functions. Instead, we used similar responses to evaluate time allocation and discrimination and used hold-down requirements to explore if the price was incorporated in the discriminative cue.

More recently and consistent with the suggestion that there are at least two projection fibers, the dorsal diencephalic conduction system and the MFB for the reward signal of the ICSS (Fakhoury et al., 2016). Trujillo-Pisanty et al. (2020) found that although dopamine neurons may contribute to both the sensitivity (input) and output (cost of the rewarding response or value of alternatives) of the reward-growth function when using optical-ICSS, it only alters the output of the reward-growth function when using electrical-ICSS. Our observation that increased prices and pimozide pretreatment induced a dose-dependent rightward shift of the reinforcement gradient but the generalization gradient is insensitive to both price or pimozide pretreatment is in full support of the RMM.

The main limitation of our study is that we do not have enough experimental data regarding the subjective experience of the −0.6 log cue stimuli used since we did not obtain a threshold curve first. We could argue that the high-strength ICS exerts a strong discriminative control producing accurate lever selection correlated with the variations of strength in the discriminative cue as evidence of subjective experience. When rats are confronted with the discriminative cue, they did not hesitate long enough to miss a reinforcer in the trial, not even when the strength in the cue was close to middle between the training cues and response prices are low; however, at the highest price, an increase in missed trials was observed. Despite the variability, a non-significant increase in missed trials can be observed when the cues lay close to the middle between training cues. However, the similarity between the DI_50_s of the generalization gradients estimated with the reinforcer obtained or holds emitted, suggests that missed trials may be related to pausing in the holds rather than hesitating to respond to a timed trial that was limited to maintain reinforcer density. If we plot correct responses to the low-strength cue, we obtain a mirror image of the high-strength gradient. However, even the accurate responses to low- and high-intensity discriminative cues and even if our stimulation parameters were within the range that rats could detect, it does not guarantee that the rats had the subjective experience with the low-strength cues. To this aim, we should obtain the threshold curve first, explore omission trials where neither the low- or high-strength cues were presented, or use a third option response as has been suggested in drug discrimination studies (Swedberg and Järbe, 1986).

Another limitation of the study is the high current used that approached saturation. Had we used a narrow range of discriminative cues, training would be prolonged, and the slope of the generalization gradient would have increased, as it happens in drug discrimination studies, but we guess similar results would be obtained. Worth exploring would be a larger range of prices to allow the fitting of the RMM to contrast some predictions of the model using both electrical and optical ICS.

## Conclusion

In conclusion, we reproduced observations that rats tradeoff response time allocation as a function of intensity or frequency; sigmoid functions were displaced to the right when long responses were required. Rats that learned the discrimination attained a discrimination index of 90–98%. Discrimination accuracy decreased slightly with the increase of hold requirement but of most importance, generalization gradients were not displaced to the right as a function of the response requirement. Preliminary data with pimozide induced a dose-dependent displacement of time-allocation gradients consistent with early observations, but it did not affect generalization gradients. These observations are in accordance with RMM that posits that dopamine neurons are involved in the reward-system gain. It is concluded that rats integrate response requirements as part of the reinforcement tradeoff function, but response cost is not integrated with the discriminative function of ICSS.

## Data Availability Statement

The original contributions presented in the study are included in the article/Supplementary Material, further inquiries can be directed to the corresponding author/s.

## Ethics Statement

The study had the approval of the Ethics Committee of the Facultad de Psicologia, Universidad Nacional Autonoma de Mexico.

## Author Contributions

DV-M designed the study, wrote the protocol, and drafted of the manuscript. BP-G and DV-L conducted curve fitting and statistical analysis. BP-G, AT-Z, and ML-G collected the data and conducted data analysis. All authors contributed to and have approved the final manuscript.

## Conflict of Interest

The authors declare that the research was conducted in the absence of any commercial or financial relationships that could be construed as a potential conflict of interest.

## Publisher’s Note

All claims expressed in this article are solely those of the authors and do not necessarily represent those of their affiliated organizations, or those of the publisher, the editors and the reviewers. Any product that may be evaluated in this article, or claim that may be made by its manufacturer, is not guaranteed or endorsed by the publisher.

## Footnotes

1
www.slipringer.com
