# Supplementary figures and images for: On the Similarity Between the Reinforcing and the Discriminative Properties of Intracranial Self-Stimulation
Source: Front Behav Neurosci. 2022 Feb 21;16:799015. doi: 10.3389/fnbeh.2022.799015 (PMC8899289; doi:10.3389/fnbeh.2022.799015)

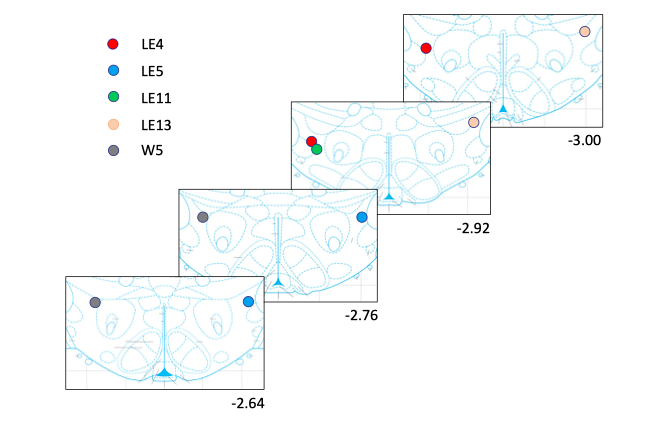

Supplement: Supplementary file 1 [file Data_Sheet_1.ZIP › ArticleData/VelazquezMartinezEtAl_Fig1.png]
