# Supplementary material for: On the Similarity Between the Reinforcing and the Discriminative Properties of Intracranial Self-Stimulation
Source: Front Behav Neurosci. 2022 Feb 21;16:799015. doi: 10.3389/fnbeh.2022.799015 (PMC8899289; doi:10.3389/fnbeh.2022.799015)
Supplement: Supplementary file 3 [file Data_Sheet_3.doc]

**Figure 1S:**

Time allocation as a function of variations of intensity and price (hold requirements). Abscissas in log scale.

**Figure 2S.A:**

Time allocation as a function of variations of intensity and price (hold requirements). Abscissas in log scale.

**Figure 2S.B:**

Time allocation as a function of variations of intensity and price (hold requirements). Abscissas in log scale.

**Figure 3S:**

Time allocation as a function of variations of frequency and price (hold requirements). Abscissas in log scale.

**Figure 4S.A:**

Time allocation as a function of variations of frequency and price (hold requirements). Abscissas in log scale.

**Figure 4S.B**

Time allocation as a function of variations of frequency and price (hold requirements). Abscissas in log scale.

**Figure 5S:**

Missed choice trials as a function of price (hold requirements) and stimulus intensity during training on free-choice trials. Mean of last 5 training sessions per subject ± S.E.

**Figure 6S:**

Discrimination Index as a function of variations of intensity and price (hold requirements). Abscissas in log scale.

**Figure 7S:**

Discrimination Index as a function of variations of frequency and price (hold requirements). Abscissas in log scale.

**Figure 8S:**

Relationship for amplitude variations of DI_50_-S^R^ and TA_50_-Hold-down.

**Figure 9S:**

Individual relationship for amplitude variations of discrimination and reinforcement parameters.

**Figure 10S.**

Effects of increasing doses of pimozide on TA_50_, TA-slope and TA-location parameters (A-C) and on DI_50_, DI-slope and DI-location (D-E). Different symbols and colors represent performance after 1, 2 or 4 s hold-down requirement. Each point is the mean (± S.E.) of 5 subjects tested twice with each dose.
